# Comprehensive knowledge of HIV prevention among fishing communities of Lake Kyoga, Uganda, 2013

**DOI:** 10.1186/s12889-020-8146-6

**Published:** 2020-01-08

**Authors:** Leocadia Kwagonza, Lilian Bulage, Paul Edward Okello, Joy Kusiima, Daniel Kadobera, Alex Riolexus Ario

**Affiliations:** 1 Uganda Public Health Fellowship Program, P.O. Box 7272, Kampala, Uganda; 2 Makerere University School of Public Health, Kampala, Uganda

## Abstract

**Background:**

Compared to the general population in Uganda, fishing communities suffer greater burden of HIV/AIDS. We determined the level of comprehensive knowledge on HIV prevention and its associated factors among fishing communities of Lake Kyoga.

**Methods:**

We conducted secondary analysis of data from the Lake Kyoga Behavioral Survey, a population-based sample survey on behavioral risk factors for HIV, syphilis, and schistosomiasis among adults in fishing communities of Lake Kyoga in 2013. We defined comprehensive knowledge as having correct knowledge on HIV prevention (consistent condom use, faithfulness, a healthy-looking person can have HIV, and HIV cannot be transmitted through food-sharing, witchcraft or handshake). We used logistic regression to determined potential factors associated with comprehensive knowledge on HIV prevention and control for confounding.

**Results:**

Of 1780 persons in the sample, 51% (911/1780) were females. The mean age was 32 (range: 15–97) years. Overall, 51% (899/1780) of persons had comprehensive knowledge on HIV prevention. Level of comprehensive knowledge on HIV prevention was similar between females (52%, 449/911) and males (49%, 450/869). Males (76%, 658/869) had lower knowledge on HIV transmission from mother to child during breast feeding compared to females (81%, 738/911) (*p*-value 0.019). Fishermen (46%,324/711) who lived > 5 km away from a health center compared to 54% (572/1066) who lived within 5 km radius were less likely to have comprehensive knowledge on HIV prevention (PRR_adj_ = 0.8; 95%CI = 0.5–0.92). Those who had ever tested for HIV were more likely to have comprehensive knowledge of HIV transmission (PRR_adj_ = 1.1; 95% 1.03–1.70).

**Conclusion:**

Half of the population of Lake Kyoga fishing community had comprehensive knowledge of HIV prevention. Long distances from health facilities reduced the level of comprehensive knowledge on HIV transmission. HIV testing increased the level of comprehensive knowledge on HIV transmission. Ministry of health should ensure that HIV/AIDS information; education and communication and HIV counseling and testing activities are intensified in fishing communities of Lake Kyoga, with more emphasis on communities living in distances of more than 5 km away from the health facility.

## Background

According to the Joint United Nations Programme on HIV and AIDS (UNAIDS), 36.7 million people were living with HIV globally by end of year 2016, yet 30% did not know their HIV status [1, 2]. By end of 2016, 1.4 million people were living with HIV/AIDS in Uganda. The adult HIV prevalence was 6.5% [3]. HIV prevalence among the fishing communities in Uganda is estimated to be three times that of the general population [4]. A study conducted in 2013 revealed 22% overall HIV prevalence in the fishing communities [4]. This high HIV prevalence among this community is attributed a number of factors including population movement, exchange of fish for sex, commercial sex, drug abuse, limited access to HIV prevention, testing services as well as poor perceptions and attitude towards HIV prevention programs among others [5].

Over four decades, the world has been fighting the HIV/AIDS epidemic with significant success stories in some countries such as Thailand [6]. In Africa, successful responses have addressed sensitive social factors surrounding HIV prevention, such as sexual behaviour, drug use, and gender equalities, countered stigma and discrimination, and mobilised affected communities; but such responses have been limited to specific areas [7].

Uganda employed a number of strategies focusing on behaviour change specifically involving promotion of faithfulness, abstinence, consistent condom use as well as sexual partner reduction which contributed to decline HIV incidence. In the late 1980s and early 1990s, HIV programs employed the health belief model using mass media messages such as daily drum-beating on the radio commonly known as “*gwangamujje*”, in conjunction with various locally developed forms such as community dramas radio talks [8]. These were important vehicles for raising awareness and fostering changes in behavioural norms. Since the beginning of fight against HIV, Uganda’s approach to behavioral change has relied primarily on community interventions using “*expert clients*”, community-based AIDS counsellors, health educators, peer educators, and other types of specialists have also been trained [9, 10]. These efforts and approaches were to improving knowledge on and hence HIV prevention to facilitate individual behaviour change.

As a result of these efforts Uganda has registered a remarkable decline in the HIV prevalence from 30% in 1991 to 6.5 in 2016 [11, 12]. Despite the significant reduction in HIV prevalence in the general population, fishing communities still experience high burden of HIV varying from 15 to 22% [13]. These communities have been characterised among populations that are at high risk for HIV infection [14]. The high HIV prevalence in the fishing communities has been attributed to factors such as high mobility, commercial sexual activities, and a lack of access to HIV prevention and testing services [4].

In-addition, fishing communities face numerous barriers in fulfilling their sexual and reproductive health rights (SRHR) [15]. They have limited access to information due to limited availability of health services that often times at a distance, high degree of mobility among fisher folks, high rates of transactional sex, the fatalistic belief that they are more likely to die fishing than of HIV, coupled with low knowledge, attitudes and practices towards HIV prevention programmes [5, 16]. A few studies have assessed HIV- related knowledge among fishing communities of Lake Victoria and risk factors for HIV transmission. In these communities, HIV knowledge was found to be around 31% [4]. In addition, activities such as tourism and cross-border trade are more prevalent unlike Lake Kyoga that is located in land. Such differences have potential to limit or improve access to information on HIV prevention. While many studies have examined how key behaviour changes (abstinence, faithfulness, and condom use) have contributed to the decline in HIV prevalence, few have studied the level of comprehensive Knowledge of HIV prevention and the associated factors among fishing communities. Yet adoption of healthy behaviours largely depends on comprehensive or accurate knowledge of HIV prevention [17]. We determined the level of comprehensive knowledge on HIV prevention and its associated factors among fishing communities of Lake Kyoga.

## Methods

This was a secondary analysis of data from the Lake Kyoga Behavioural Survey of August 2013. The Lake Kyoga Behavioral Survey, was a population-based survey on behavioral risk factors and sero-status for HIV, syphilis, and schistosomiasis among adults in the fishing communities of Lake Kyoga in 2013. The target population comprised of residents of the Lake Kyoga landing sites that largely depend on the harvest or processing of fishery resources to meet their welfare needs. The survey was conducted in eight districts of Amolator, Apac, Buyende, Dokolo, Kaberamaido, Kaunga, Serere and Nakasongola with the aim of providing baseline data for future surveys. The main survey was conducted among adult Ugandans aged ≥15 years. Residents who had stayed in Lake Kyoga community for at least 3 months with the ability to communicate in at least one of the four study languages (English, Luganda, Ateso, Luo) were included in the study. The questionnaire were translated into the study languages above.

The sampling was done by stratifying two landing sites that is fish handling and non-fish handling facilities. This was so because sites that have a fish handling facility tend to attract more organized fish transactions, are more permanent and recognized by both large-scale traders and authorities. Their linkages with the outside markets are more visible. The mobility and external exposure of such facilities may have a bearing on the HIV dynamics in this setting. On the other hand, non-fish landing sites do trade fish on the anchored boat, canoe or on the ground, and their trade activities are less predictable.

At the time the survey was conducted, there were 139 landing sites around Lake Kyoga and 22% [18] had fish handling facilities. This provided a basis for the probability proportionate to size (PPS) sampling of the sites. The sample therefore had eight landing sites with fish handling facilities and 32 that were without fish handling facilities. From each of the 40 pre-selected landing sites, 22 households were selected using systematic random sampling. A household was defined as a domestic unit consisting of an individual or a group of individuals who share the same living accommodation (house or hut), irrespective of whether or not they shared the same family life. The estimated sample was weighted to cater for level of precision, non-response, average household size, HIV prevalence and probability of selection since large landing sites would have more chances of being selected than smaller ones. Large landing sites were additionally sampled by a factor of an additional 22 households as reflected by Probability Proportionate to Size methodology. The methods for the main study are described elsewhere [19].

Our outcome variable was comprehensive knowledge on HIV prevention which was computed based on the number of people who accurately answered the three knowledge questions that consistent use of condom during sexual intercourse, being faithful, knowing that a healthy-looking person can have HIV, and rejecting the three most common misconceptions about HIV prevention*.* The three common misconceptions of HIV transmissions considered were; HIV cannot be transmitted through sharing food, witchcraft, and shaking hands w*i*th a HIV infected person [12].

For the Independent variables, we grouped the independent variables into demographic and health system related factors. Demographic variables included: age, gender, education, religion, tribe, district of residence, and marital status. Health system related factors included: distance from health facility based on the recommended 5 km radius according to the Health Sector Strategic and Investment Plan (HSSIP) [20], and ever tested for HIV which was determined through self-reports.

We analyzed data using STATA version 13.0. For the outcome variable, all the six questions had to be answered correctly for a respondent to have comprehensive knowledge of HIV prevention. Since the estimated sample was weighted to cater for probability of selection, level of precision, non-response and average household size, we did not weigh the data at analysis since the estimated sample size was weighted at sample size calculation. Using logistic regression, we identified independent variables that were associated with comprehensive knowledge of HIV prevention. At bivariate analysis level, each independent variable was tested for association with comprehensive knowledge of HIV prevention. Associations with a *p* value < 0.2 at bivariate analysis level were considered for inclusion in the multivariable model. Any other known potential confounders such as age and sex from literature were also added to the model. The model was built using forward stepwise elimination method. In the final multivariable model, associations with *p* value < 0.05 were considered statistically significant and therefore independently associated with the outcome. To asses for confounding, we considered a 10% change in the prevalence risk ratio upon addition of variables to the multivariable model. We tested for interaction between variables such as age-gender, age-education and distance from the health facility, using the log-likelihood ratio test.

## Results

A total of 1780 respondents were recruited in the main study. Therefore this secondary data analysis was based on responses from 1780 respondents. The mean age was 32 years (SD 12) ranging from 15 to 97 years. Fifty one percent (911/1780) of the study participants were females (Table 1).

**Table 1:** Socio-demographic characteristics of the study participants

Variable	Frequency (*n* = 1780)	Per cent age (%)
Sex		
Males	869	49
Females	911	51
Age		
< 20	265	15
20–29	541	30
30–39	515	29
40–49	318	18
50+	141	9
Religion		
Catholic	586	33
Anglican	705	40
Pentecostal	193	11
Muslim	237	13
Others	59	3.3
Tribe		
Etesots	299	17
Musoga	177	9.9
Kumam	85	4.8
Langi	561	32
Ganda	90	5.1
Acholi	21	1.2
Others	547	31
Education		
No formal education	139	7.8
Primary	1246	70
Secondary and above	395	23
Marital status		
Single never married	341	19
Married/ cohabiting	1228	69
Single for other reasons^a^	211	12

^a^divorced, widowed or separated

Overall, 51% (899/1780) of the respondents had comprehensive knowledge on HIV prevention. The level of comprehensive knowledge on HIV prevention was similar among females 52% (449/911) and males 49% (450/869) (Table 2).

**Table 2:** Knowledge and misconceptions of HIV transmission among fishing communities of Lake Kyoga, 2013

Knowledge questions	Overall % (*n* = 1780)	Male % (*n* = 869)	Female % (*n* = 911)
Knowledge of HIV prevention methods			
HIV can be prevented by consistent use of condom during sexual intercourse,	87	87	87
HIV can be prevented by being faithful,	90	90	89
knowing that a healthy-looking person can have HIV	89	90	87
Misconceptions around HIV transmission			
HIV cannot be transmitted through sharing food,	86	87	85
HIV is not transmitted through witchcraft	83	84	82
HIV is not transmitted through shaking hands w*i*th a HIV infected person	81	83	79
Comprehensive knowledge of HIV transmission	51	49	52

**Fig. 1:**
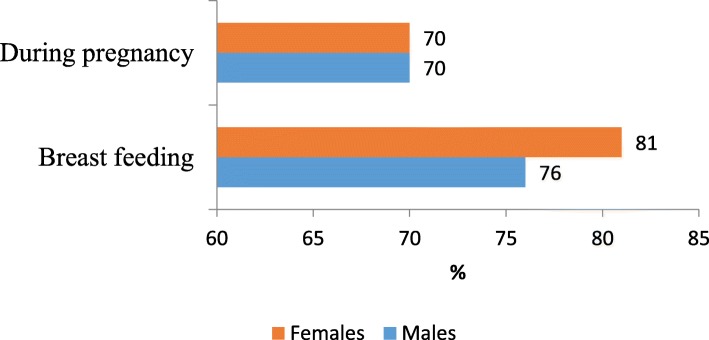
Knowledge of Mother-to- Child HIV transmission among fishing communities of Lake Kyoga, 2013

The level of knowledge on prevention of mother-to-child transmission was similar among the males and females. The level of knowledge of HIV transmission to the unborn child or during pregnancy was the same between males and females at 70% (1246/1780). Similarly, 81% (738/911) of females and 79% (687/869) of males knew that HIV can be transmitted to the baby through breast feeding (Fig. 1).

The level of knowledge on HIV prevention methods was similar among males and females. Ninety per cent of males (782/869) and 87% (793/911) of females knew that HIV can be prevented through condom use, while 90% (782/869) males and 89% (811/911) females knew that HIV can be prevented through condom use (Table 2). There was no difference in the levels of knowledge on misconceptions around HIV transmission among males and females. Eighty seven percent of males (756/869) and 85% (/774/911) of females knew that HIV cannot be transmitted through shaking hands with an infected person. Also, 84% (730/869) of males and 82% (747/911) of females knew that a person cannot get HIV through witchcraft practices. Similarly, 83% (720/869) of males and 79% (720/911) of females knew that HIV cannot be transmitted through sharing food with an infected person (Table 2).

At bivariate analysis level, comprehensive knowledge on HIV prevention was associated with distance to health facility (CPRR = 0.8; 95%CI: 0.81–0.94), having attained at least secondly level education (CPRR = 2.5, 95% CI: 1.9–2.3), age-group 20–29 (CPRR = 1.4; 95% CI: 1.13–1.68), and having ever tested for HIV (CPRR = 1.5, 95% CI: 1.2–1.9). The relationship between gender and comprehensive knowledge of HIV prevention was not statistically significant (CPRR = 1.0, 95% CI: 0.95–1.14) (Table 3).

**Table 3:** Factors associated with comprehensive knowledge of HIV transmission among fishing communities of Lake Kyoga, 2013

Variable	Have comprehensive knowledge of HIV transmission		CPR	95% CI	APR	95% CI
	No (*n*=881)	Yes (*n* = 899)				
Gender						
Female	462 (51)	449 (49)				
Male	419 (48)	450 (52)	1.0	0.95–1.14	1.01	0.80–1.32
Marital status						
Single never married	179 (52)	162 (48)				
Married or living with partner	584 (47)	644 (52)	1.2	0.97–1.41		
Single for other reasons	118 (56)	93 (44)	0.95	0.83–1.08	–	
Age						
< 20	150	115	1.0		1.0	
20–29	242	299	1.4	1.13–1.68	1.3	1.13–1.19
30–39	248	267	1.2	1.03–1.53	1.0	0.92–1.51
40–49	164	154	1.1	0.93–1.34	0.9	0.87–1.42
50+	77	64	1.0	0.89–1.19	0.9	0.82–1.15
Religion						
Catholic	299 (51)	287 (49)				
Muslims	105 (44)	132 (56)	1.0	0.99–1.8		
Anglican	354 (50)	351 (50)	1.0	0.90–1.15		
Others	165 (56)	129 (43)	0.93	0.85–1.02		
Educational level						
No formal Education	127	54	1.0		1.0	
Primary level	644	602	2.0	1.5–2.7	1.5	1.1–2.3
Secondary and above	152	243	2.5	1.9–3.3	1.9	1.2–3.2
Distance to Health facility						
≤ 5 km	494	572	1.0		1.0	
> 5 km	387	324	0.8	0.81–0.94	0.8	0.5–0.92
Ever tested for HIV						
No	237	177	1.0		1.0	
Yes	644	722	1.4	1.1–1.6	1.1	1.02–1.70

CPRR refers to the crude prevalence ratio, APRR refers to adjusted prevalence ratio

After adjusting for potential confounders, four factors: age-group 20–29, having attained at least secondary level education, distance to Health facilities, and having ever tested for HIV were associated with comprehensive knowledge of HIV transmission. We found that 55%(299/541) respondents of age-group 20–29 were more likely to have comprehensive knowledge on HIV prevention compared to 43% (115/265) of young respondents of less than 20 years (APRRR = 1.3; 95% CI:1.13–1.19). Sixty one percent (243/395) of respondents with at least secondary level education were two times more likely to have comprehensive knowledge on HIV prevention compared to 30% (54/181) of those with no formal education (APRR = 1.9, 95% CI: 1.2–3.2), while 46% of respondents(324/711) who lived > 5 km away from a health centre compared to 54% (572/1066) who lived within 5 km radius were less likely to have comprehensive knowledge on HIV prevention (APRR = 0.8; 95%CI = 0.5–0.92) (Table 3). There was no interaction between age-gender, age-education or education -distance from the health facility.

## Discussion

Half of the participants in the Lake Kyoga fishing community had comprehensive knowledge of HIV prevention. People; aged 20–49, who had ever tested for HIV and those with at least Secondary level education were more likely to have comprehensive knowledge on HIV prevention. People who lived beyond 5 km away from health facilities were less likely to have comprehensive knowledge of HIV prevention.

The Lake Kyoga fishing community had higher levels of comprehensive knowledge on HIV prevention compared to the general population of 39.5% as of 2016 [12]. The higher comprehensive knowledge could be attributed to the increase and intensification of interventions targeting the most at risk populations such as fishing communities. Interventions such as moonlight clinics that offer HIV counselling and testing services at night to the convenience of various categories of populations that are at risk of HIV and engage in busy work schedules throughout the day, pre-exposure prophylaxis, test and treat and behaviour change communications strategies among others have been successfully implemented in these key populations [21]. Such interventions have the potential to improve community level comprehensive knowledge of HIV transmission.

In this study, knowledge on prevention of mother to child transmission was relatively high among both males and females. This can be attributed to the fact that, prevention of mother to child HIV transmission gained a lot of attention and support from both the government and development partners. For example, in 2001 the United Nations General Assembly Special Session (UNGASS) on HIV/AIDS proposed a 50% reduction in proportion of HIV infected infants by 2010 [22]. In response, the World Health Organization and its U.N. partners have been promoting four main approaches to the prevention of mother-to-child transmission (PMTCT) of HIV/AIDS [23]. The four main approaches include prevention of HIV infection among women of childbearing age, prevention of unintended pregnancies in HIV-infected women by enhancing their contraceptive use, elimination of mother-to-child HIV transmission and provision of care and support for HIV-infected mothers and their children [23, 24]. These strategies have been adapted and implemented in all HIV control programs and HIV health related messages are modified around these four pillars. This has consequently improved knowledge of prevention of mother to child HIV transmission in the population. In addition, development and implementation of guidelines on HIV prevention targeting key populations have contributed to the spread of knowledge among vulnerable populations [25].

It has been documented that education plays a significant role in determining one’s social status, and in many cases, it translates to better occupation, income and access to information [26]. This study found education to be a significant predictor of having comprehensive knowledge of HIV prevention, a finding consistent with those of the 2007 Kenya AIDS Indicator Survey (KAIS) and a study conducted among Malawian women [27]. Formal education has the ability to influence some’s opportunities to access information on HIV and AIDS. Subsequently the acquired information can be used to protect themselves from infection, and also motivate them to take better care of their health.

In this study, respondents that lived in close proximity to health centres were more likely to have comprehensive knowledge of HIV compared to respondents that lived more than 5 km radius. This is probably because those that lived near to the health facilities had access to HIV related information which enhanced their knowledge. These findings are consistent with a study conducted in Mozambique where those in close proximity to health facility offering ART had higher knowledge of HIV transmission [28].

We also found that history of ever tested for HIV was associated with comprehensive knowledge on HIV prevention. The association between history of ever tested for HIV and comprehensive knowledge on HIV prevention could be attributed to the fact that people who go for HIV testing are given health information in form of counselling sessions before and after the test and this is a requirement of the national HIV testing in Uganda [18]. HIV counselling and testing services provide information on HIV transmission, prevention, and how to live positively in case of positive results.

## Limitations

This was a population based survey on a large population of fishing communities therefore findings are representative of the population in this community. Also, the Lake Kyoga Behavioral Survey whose date we utilized in this study used self-reports of knowledge on HIV prevention. This might have introduced respondent social desirability bias. Therefore, it is possible that we overestimated the level of comprehensive knowledge on HIV prevention in this community. Also, the authors of the primary study stated that the estimated sample was weighted to cater for probability of selection, level of precision, non-response, average household size, HIV prevalence as in the methodology. No more details were provided. Therefore we did not weigh the sample again at analysis as this would be double weighting.

## Conclusion

Half of the population of Lake Kyoga fishing community had comprehensive knowledge of HIV prevention. People; aged 20–49, who had ever tested for HIV and those with at least secondary level education were more likely to have comprehensive knowledge on HIV prevention. People who live beyond 5 km away from health facilities were less likely to have comprehensive knowledge of HIV prevention. The findings from this study can be generalised to fishing communities that have similar settings as Lake Kyoga.

## Recommendations

Ministry of Health should ensure that HIV/AIDS information; education and communication and HIV counselling and testing activities are intensified in fishing communities of Lake Kyoga, with more emphasis on communities living in distances of more than 5 km away from the health facility. Further research should be conducted to investigate challenges faced by fishing communities especially of Lake Kyoga in accessing knowledge on HIV prevention.

## Data Availability

The data that support the findings of this study are available from the US Centers for Disease Control and Prevention-Uganda but restrictions apply to the availability of these data and so are not publicly available. Data are however available from the corresponding author upon reasonable request and with permission of the US Centers for Disease Control and Prevention-Uganda.

## Abbreviations

AIDS: Acquired Immune Deficiency Syndrome
APRR: Adjusted Prevalence Risk Ratio
ARV: Antiretroviral
CI: Confidence Interval
CPRR: Crude Prevalence Risk Ratio
HIV: Human Immune Virus
HSSIP: Health Sector Strategic and Investment Plan
KAIS: Kenya AIDS Indicator Survey
PMTCT: Prevention of Mother-to-Child Transmission
SRHR: Sexual and Reproductive Health Rights
UNAIDS: United Nations Programme on HIV and AIDS
UNGASS: United Nations General Assembly Special Session
VCT: Voluntary Counselling and Testing
